# Balance Confidence and Falls in Individuals With Charcot–Marie–Tooth Disease: A Cross‐Sectional Observational Study

**DOI:** 10.1002/hsr2.70682

**Published:** 2025-04-21

**Authors:** Kirsten M. Anderson, Bopha Chrea, Riccardo Zuccarino, Michael E. Shy, Jason M. Wilken

**Affiliations:** ^1^ Department of Physical Therapy and Rehabilitation Science, Carver College of Medicine University of Iowa Iowa City Iowa USA; ^2^ Department of Orthopedics and Rehabilitation, Carver College of Medicine University of Iowa Iowa City Iowa USA; ^3^ Department of Neurology, Carver College of Medicine University of Iowa Iowa City Iowa USA

**Keywords:** ankle foot orthosis, balance, Charcot–Marie–Tooth disease, falls

## Abstract

**Background and Aims:**

Impaired balance and functional deficits are common in individuals with Charcot–Marie–Tooth disease (CMT). Many individuals with CMT use ankle foot orthoses (AFOs) to improve their balance and function. The aim of this study was to evaluate the fall frequency, perceived effect of AFOs on balance, and balance confidence of individuals with CMT who currently use AFOs.

**Methods:**

Three hundred and six individuals participated in this study. Questions related to fall frequency, questions about the perceived effect of AFOs on balance, and the Activities Specific Balance Confidence Scale were distributed to individuals with CMT via e‐mail using a Patient Contact Registry.

**Results:**

Many participants reported falling in the preceding 24 h (14% of participants) or week (38% of participants). 78% of participants indicated their AFOs improve their balance. Participants reported decreased balance confidence across a range of activities, with highest confidence for level ground walking and standing tasks (> 60%), markedly decreased confidence when walking on stairs, slopes, or with external stimuli (40–55%), and poor confidence when walking on icy sidewalks, when bumped, or on unsteady surfaces (< 40%).

**Conclusions:**

The data presented here provides insight into the frequency of falls and balance confidence for individuals with CMT who use AFOs. The Activities Specific Balance Confidence Scale can be used as an assessment tool to identify CMT patients with low balance confidence who are at risk for falls. This information can be used to target patient education and tailor treatment plans and interventions to address challenging activities. Further, this study will help to focus future studies investigating the effects of AFO design on balance confidence and falls.

## 1 Introduction

Charcot–Marie–Tooth disease (CMT) is one of the most common inherited peripheral neuropathies that affects the motor and sensory axons of the peripheral nervous system [1]. Depending on the axons affected, motor and sensory function is progressively impaired, typically beginning in the longest axons and progressing proximally over time. Affected patients typically have progressive distal weakness, muscle atrophy, and sensory loss, first in the feet and lower legs, followed by the hands [2, 3, 4, 5]. Many researchers have identified muscle weakness and loss of proprioception as primary causes of impaired balance and decreased function in individuals with CMT [6, 7, 8, 9]. Impaired balance and function put individuals at increased risk for falls, fall‐associated injuries, and limited participation due to fear of falling [10]. Many individuals with CMT use ankle foot orthoses (AFOs) to improve gait, function, and balance, but little is known about balance confidence and fall risk in AFO users.

Impaired balance, decreased balance confidence, and increased fall risk are common in individuals with a range of neurological conditions that impair strength and sensation [10, 11, 12, 13, 14]. For example, balance confidence has been associated with the likelihood of falling in individuals with diabetic peripheral neuropathy [13] and in a broad sample of individuals with neurologic conditions falls were associated with disturbed gait, poor balance, and fear of falling [14]. In one study, 86% of individuals with CMT cited muscular weakness as a cause of their fall [10]. However, specific data regarding fall frequency, balance confidence, and the effect of AFOs for individuals with CMT is limited. More robust data is needed to inform AFO design and development, to establish targeted rehabilitative interventions, and improve patient education. The primary purpose of this study was to survey a large cohort of individuals with CMT who currently use AFOs, to assess balance confidence, fall frequency, and the perceived effects of AFOs on balance. We hypothesized that individuals with CMT who use an AFO would fall frequently and would have impaired balance confidence but would perceive their AFOs as being beneficial to their balance.

## 2 Methods

The study was approved by the University of Iowa Institutional Review Board (IRB‐01) and utilized a web‐based Patient Contact Registry to distribute the study survey by email, in a manner similar to previous studies [15, 16]. This study was determined to be exempt from informed consent requirements, and participants were provided an overview with the study and were invited to complete the study if they met the inclusion criteria.

A single introductory email was sent to all individuals in the Contact Registry with a link to the online survey that included questions concerning fall frequency, questions about the perceived effect of AFOs on balance, and the Activities Specific Balance Confidence Scale (ABC) [17]. Individuals between the ages of 18 and 90, who had been diagnosed with CMT and who use AFOs were invited to participate in this study. Participation in the study was not limited by the frequency of AFO use. Data regarding age, biological sex, height, weight, ethnicity, and CMT type were collected.

Individuals in the Patient Contact Registry have a similar distribution of CMT type to those participating in natural history studies; 53% of registry participants have CMT1A, 3.8% have CMT1B, 9.7% have CMT2A, 2.0% have CMT4, 4.9% have CMTX, and 26% have CMT without a subtype or genetic classification. Nearly 3500 individuals participate in the registry; however, the proportion of those who use AFOs is unknown.

Participants were asked questions concerning how frequently they had fallen in the last 24 h and in the last week and were asked to rank how their AFOs affect their balance from 0 = impairs balance to 10 = improves balance. The ABC survey has been used in multiple patient populations and is a quick and easy method of assessing balance confidence across 16 functional activities [11, 12, 13, 17, 18]. The ABC takes approximately 3 min to complete, is widely used in rehabilitation, and has psychometric evidence to support its use for quantifying balance confidence in neurologic diseases [11, 12]. Participants rate their confidence level in performing each task using a scale of 0–100, with 0 = no confidence and 100 = complete confidence.

Mean and standard deviations, as well as percent populations, were calculated for patient demographic and clinical characteristics. Mean and standard deviations, percent populations, median, and quartile values are presented for balance confidence and falls data. Data analysis was completed using SPSS v29 (IBM, New York, NY).

## 3 Results/Findings

A total of 306 individuals completed the falls and balance‐related questions and ABC questionnaire. This accounts for approximately 10% of individuals in the Inherited Neuropathy Consortium (INC) Patient Contact Registry. The total number of individuals in the INC Patient Contact Registry who use AFOs is unknown, therefore a true response rate cannot be calculated. Approximately 58% of responders were female, and the average age (SD) was 57.2 (14.8) years. The INC consists of 49% females with an average (SD) age of 49 (21.3) years. Additional demographic and clinical characteristics of the participants are shown in Table 1.

**Table 1:** Demographic and descriptive data from 306 study participants.

Mean (SD) or % study participants	
Characteristic	
Age (years)	57.2 (14.8)
Female (%)	58
Height (cm)	168.6 (11.4)
Weight (kg)	78.7 (22.1)
Geographic location (%)	
North America	83
Europe	14
Asia	1
Australia	1
South America	0
Africa	0
Type of CMT (%)	
CMT 1A/A1	42
CMT 1B/B1	4
CMT 2A	5
CMT 4	0
CMT X	2
Other	32
Unknown	14

**Figure 1:**
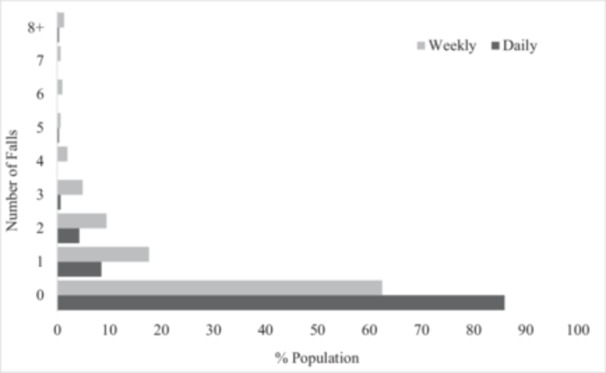
Frequency of falls on daily and weekly basis.

**Figure 2:**
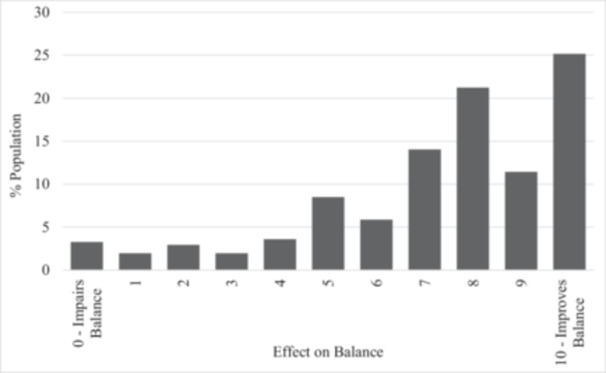
Reported effect of AFOs on participant's balance. Rating of 0 indicates that AFO impairs their balance, rating of 10 indicates that AFOs improve their balance.

Participants reported the median (1st quartile, 3rd quartile) number of falls they had experienced in the last 24 h and in the last week (Figure 1), and how their AFOs effect their balance (Figure 2). A total of 14% of participants indicated falling in the last 24 h with a median number of 0.0 (0.0, 0.0) falls, while 38% of participants indicated they fell a median of 0.0 (0.0, 1.0) times in the last week (Table 2). Individuals who reported falling in the last 24 h had an average balance confidence of 38% (16%) while those who reported no falls had an average balance confidence of 48% (22%). Individuals who reported falling in the last week had an average balance confidence of 43% (21%), compared to an average balance confidence of 49% (22%) for those who reported no falls. 78% of respondents indicated that their AFOs improve their balance, 8.5% indicate their AFOs have no effect on their balance, and 14% indicate they impaired their balance. The median reported effect of AFO use on balance was 8.0 (6.0, 10.0), with larger values indicating an improvement in balance.

**Table 2:** Breakdown of activities based on reported confidence level from highest to lowest, falls reported in the last 24 h and week, and impact of AFO use on balance.

	Mean (SD)	25 quartile	Median	75 quartile
**Highest confidence**				
…reach for a small can off a shelf at eye level?	75 (26)	60%	90%	90%
…get into or out of a car?	73 (25)	60%	80%	90%
…walk around the house?	68 (28)	50%	80%	90%
…walk outside the house to a car parked in the driveway?	66 (29)	48%	70%	90%
…sweep the floor?	61 (31)	30%	70%	90%
…walk across a parking lot to the mall?	58 (31)	30%	60%	83%
**Moderate confidence**				
…walk up or down a ramp?	52 (31)	30%	50%	80%
…bend over and pick up a slipper from the front of a closet floor?	50 (30)	20%	50%	80%
…walk in a crowded mall where people rapidly walk past you?	47 (31)	20%	45%	70%
…step onto or off an escalator while you are holding onto a railing?	45 (32)	20%	40%	70%
…walk up or down stairs?	43 (30)	10%	40%	70%
**Lowest confidence**				
…are bumped into by people as you walk through the mall?	35 (29)	10%	30%	60%
…step onto or off an escalator while holding onto parcels such that you cannot hold onto the railing?	21 (27)	0%	10%	30%
…stand on your tiptoes and reach for something above your head?	19 (28)	0%	0%	30%
…stand on a chair and reach for something?	19 (26)	0%	10%	30%
…walk outside on icy sidewalks?	12 (20)	0%	0%	13%
**Falls**				
In the last 24 h	0.2 (0.9)	0.0	0.0	0.0
In the last week	0.9 (2.5)	0.0	0.0	1.0
Effect of AFOs on balance	7.3 (2.6)	6.0	8.0	10.0

The median (1st quartile, 3rd quartile) balance confidence across all activities was 50% (10%, 80%), demonstrating decreased and highly varied balance confidence. The activity with lowest average balance confidence was walking outside on icy sidewalks, 0.0% (0.0%, 13%), and the activity with highest average balance confidence was reaching for a small can off a shelf at eye level, 90% (60%, 90%).

Activities were divided into three groups based on percent balance confidence ratings (Table 2). Walking and standing activities generally had the highest average balance confidence with values over 55%. Walking on unlevel surfaces, using stairs or elevators, walking near others, or bending over showed moderate balance confidence with values between 40% and 55%. Activities with perturbations or changes to the standing or walking surface had the lowest balance confidence below 40%.

## 4 Discussion

The primary purpose of this study was to survey a large cohort of individuals with CMT who use AFOs to assess balance confidence, fall frequency, and perceived effect of AFOs on balance. The findings of this study corroborate pilot work completed by others [19] and highlight the persistent negative effects of CMT on fall frequency and balance confidence, despite the perceived benefits of AFO use on balance.

While 77% of participants indicated that their AFOs improved their balance, 14% of participants reported falling within the last 24 h and 37% within the last week. The frequency of daily and weekly falls in this study is more than double the rate observed in a cross‐sectional study by Ramdharry et al. [10] These substantially higher rates of falls demonstrates the need to identify and address balance deficits in individuals with CMT who use AFOs, because impaired walking function is the most significant contributor to reduced quality of life [19].

Participants in the Ramdharry et al. study were on average 7 years younger than participants in this study, and participation in that study was not limited to individuals who require an AFO [10]. Because CMT is progressive and AFOs are typically used to address significant impairment in strength and sensation [20], the higher fall rates here are likely indicative of a more impaired population and reflect the high fall risk of individuals seen in orthotics clinics. Ramdharry et al. did not specifically report the rate of AFO use in their sample, but muscular weakness (legs failing to move sufficiently, foot drop, or joints giving way), trips, slippery or uneven terrain, loss in balance, or specific ankle instability were identified as the probable causes of the reported falls [10]. Nearly two‐thirds of the reported falls occurred while walking, with many resulting in injury [10]. The ABC data presented here align with these findings and demonstrate the importance of context, such as distractions and walking surface, on balance confidence, and likely fall risk, during walking.

The highest average balance confidence was observed for common daily activities such as walking on level surfaces and standing and reaching, and moderate confidence was observed for less frequent activities that are important to general community activity, including walking on stairs or unlevel surfaces. The lowest balance confidence was reported for activities involving perturbations or changes to the standing or walking surface. The results from this study are consistent with available data from populations that have known fall risk, which also demonstrate impaired balance confidence [13, 17, 18]. Individuals with diabetic peripheral neuropathy had a similar average balance confidence score across all activities [13] and adults over 65 [17], and outpatient physical therapy patients [18] ranked the activities from highest to lowest balance confidence in a similar manner (Table 3). Although individuals may choose to avoid difficult activities, some may be unavoidable based on the geographic location (walking on icy sidewalks) or the demographics (bumped into by people) of where they live. Further, recent literature suggests that impaired balance confidence may negatively impact engagement and community participation, limiting the ability of individuals to activity engage in important activities [21, 22].

**Table 3:** Rankings of ABC results from this study and studies conducted in adults over 65 by Powel and Myers [17] and outpatient physical therapy patients by Wang et al. [18].

Balance confidence rankings from 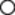 no confidence to 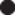 complete confidence		
Study Results	Powel and Myers [17]	Wang et al. [18]
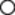 …walk outside on icy sidewalks?	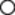 …walk outside on icy sidewalks?	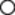 …walk outside on icy sidewalks?
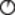 …stand on a chair and reach for something?	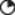 …step onto or off an escalator while holding onto parcels such that you cannot hold onto the railing?	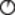 …stand on a chair and reach for something?
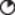 …stand on your tiptoes and reach for something above your head?	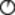 …stand on a chair and reach for something?	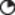 …step onto or off an escalator while holding onto parcels such that you cannot hold onto the railing?
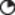 …step onto or off an escalator while holding onto parcels such that you cannot hold onto the railing?	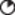 …stand on your tiptoes and reach for something above your head?	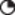 …are bumped into by people as you walk through the mall?
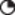 …are bumped into by people as you walk through the mall?	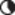 …step onto or off an escalator while you are holding onto a railing?	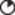 …stand on your tiptoes and reach for something above your head?
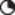 …walk up or down stairs?	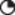 …are bumped into by people as you walk through the mall?	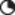 …walk up or down stairs?
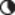 …step onto of off an escalator while you are holding onto a railing?	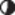 …walk up or down a ramp?***	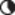 …step onto of off an escalator while you are holding onto a railing?
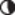 …walk in a crowded mall where people rapidly walk past you?	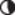 …walk in a crowded mall where people rapidly walk past you?	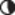 …walk in a crowded mall where people rapidly walk past you?
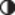 …bend over and pick up a slipper from the front of a closet floor?	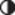 …bend over and pick up a slipper from the front of a closet floor?	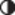 …bend over and pick up a slipper from the front of a closet floor?
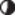 …walk up or down a ramp?	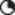 …walk up or down stairs? ***	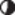 …walk up or down a ramp?
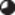 …walk across a parking lot to the mall?	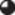 …sweep the floor?	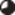 …walk across a parking lot to the mall?
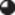 …sweep the floor?	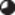 …walk across a parking lot to the mall?	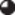 …sweep the floor?
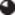 …walk outside the house to a car parked in the driveway?	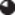 …walk outside the house to a car parked in the driveway?	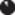 …walk around the house?
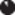 …walk around the house?	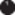 …get into or out of a car?	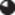 …walk outside the house to a car parked in the driveway?
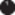 …get into or out of a car?	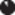 …walk around the house?	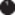 …get into or out of a car?
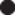 …reach for a small can off a shelf at eye level?	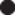 …reach for a small can off a shelf at eye level?	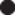 …reach for a small can off a shelf at eye level?

***Ranking differed by more than two spots from study results.

Documenting falls and ABC questionnaire results may prove useful in optimizing rehabilitative and orthotic care in an individualized matter. In the clinical setting, patients are often assessed while walking on level and flat surfaces where they are most confident and stable, while the results here suggest evaluation, AFO tuning, and treatment interventions focusing on activities that are difficult for the individual is warranted. Deficits that cannot be addressed through orthotic interventions alone may benefit from supplemental rehabilitative interventions to optimize strength and functional ability during AFO use [23]. For example, individuals who indicate poor balance confidence while walking in crowded areas may benefit from additional training to develop the skills necessary to adjust to perturbations in real‐life scenarios [24, 25, 26, 27]. Further, multiple AFO design characteristics have been linked to patient dissatisfaction with their AFO, and it is likely that specific types or designs of AFOs may best improve balance and fall risk in individuals with CMT [16, 24, 25, 26, 27]. The current understanding of the interaction between orthotic interventions and rehabilitative interventions and falls is limited.

This study includes limitations due to its reliance on the distribution of an online survey using the INC Patient Contact Registry. This includes the inability to calculate precise response rates, collect detailed information concerning AFO type or design and prior rehabilitative interventions, or assess CMT‐associated impairment. The total number of individuals in the INC Patient Contact Registry who use AFOs is unknown, therefore a true response rate cannot be calculated. Further, the inclusion of only individuals who use an AFO may have biased results toward individuals who perceive their AFOs as beneficial. However, the included sample of 306 individuals with CMT who use AFOs is by far the largest cohort to date. While these limitations may influence the interpretation and generalizability of the data, the overall results are consistent with clinical experience, provide valuable insight into the experience and opinions of several hundred individuals with CMT who use AFOs, and will provide foundational information to guide further study.

The data presented here provides insight into the frequency of falls and identifies activities associated with impaired balance confidence. Study results highlight the importance of assessing balance confidence in individuals with CMT who use AFOs and addressing low balance confidence to prevent falls. The ABC can be used to identify individuals with CMT who have low balance confidence and are at risk for falls. This information can be used to target patient education and tailor treatment plans and interventions to address challenging activities. Additional work is required to determine if specific impairments or device characteristics which correlate with low balance confidence and falls in individuals with CMT who use AFOs and determine if tailored orthotic care and balance‐specific training programs improve outcomes.

## Author Contributions

**Kirsten M. Anderson:** data curation, formal analysis, investigation, methodology, project administration, visualization, writing – original draft, writing – review and editing. **Bopha Chrea:** project administration, supervision, visualization, writing – review and editing. **Riccardo Zuccarino:** conceptualization, methodology, project administration, resources, supervision, writing – original draft. **Michael E. Shy:** conceptualization, funding acquisition, project administration, resources, supervision, writing – review and editing. **Jason M. Wilken:** conceptualization, funding acquisition, methodology, project administration, resources, supervision, validation, visualization, writing – original draft, writing – review and editing.

## Conflicts of Interest

The authors declare no conflicts of interest.

## Transparency Statement

The lead author Kirsten M. Anderson affirms that this manuscript is an honest, accurate, and transparent account of the study being reported; that no important aspects of the study have been omitted; and that any discrepancies from the study as planned have been explained. All authors have read and approved the final version of the manuscript. Kirsten M. Anderson had full access to all of the data in this study and takes complete responsibility for the integrity of the data and the accuracy of the data analysis.

## Data Availability

The data that support the findings of this study are available from the corresponding author upon reasonable request.
